# Investigating mechanisms underpinning the detrimental impact of a high-fat diet in the developing and adult hypermuscular myostatin null mouse

**DOI:** 10.1186/s13395-015-0063-5

**Published:** 2015-12-07

**Authors:** Antonios Matsakas, Domenick A. Prosdocimo, Robert Mitchell, Henry Collins-Hooper, Natasa Giallourou, Jonathan R. Swann, Paul Potter, Thomas Epting, Mukesh K. Jain, Ketan Patel

**Affiliations:** Centre for Cardiovascular & Metabolic Research, Hull York Medical School, University of Hull, Hull, UK; Case Cardiovascular Research Institute and Harrington Heart & Vascular Institute, Department of Medicine, Case Western Reserve University School of Medicine and University Hospitals Case Medical Center, Cleveland, USA; School of Biological Sciences, University of Reading, Reading, RG6 6UB UK; Department of Food and Nutritional Sciences, School of Chemistry, Food and Pharmacy, University of Reading, Reading, UK; Mammalian Genetics Unit, MRC Harwell, Oxford, UK; Institute for Clinical Chemistry and Laboratory Medicine, Universitat klinikum, Freiburg, Germany; Freiburg Institute for Advanced Studies, University of Freiburg, Freiburg, Germany

**Keywords:** Muscle, Obesity, High-fat diet, Metabolism, Myostatin

## Abstract

**Background:**

Obese adults are prone to develop metabolic and cardiovascular diseases. Furthermore, over-weight expectant mothers give birth to large babies who also have increased likelihood of developing metabolic and cardiovascular diseases. Fundamental advancements to better understand the pathophysiology of obesity are critical in the development of anti-obesity therapies not only for this but also future generations. Skeletal muscle plays a major role in fat metabolism and much work has focused in promoting this activity in order to control the development of obesity. Research has evaluated myostatin inhibition as a strategy to prevent the development of obesity and concluded in some cases that it offers a protective mechanism against a high-fat diet.

**Methods:**

Pregnant as well as virgin myostatin null mice and age matched wild type animals were raised on a high fat diet for up to 10 weeks. The effect of the diet was tested on skeletal muscle, liver and fat. Quantitate PCR, Western blotting, immunohistochemistry, in-vivo and ex-vivo muscle characterisation, metabonomic and lipidomic measurements were from the four major cohorts.

**Results:**

We hypothesised that myostatin inhibition should protect not only the mother but also its developing foetus from the detrimental effects of a high-fat diet. Unexpectedly, we found muscle development was attenuated in the foetus of myostatin null mice raised on a high-fat diet. We therefore re-examined the effect of the high-fat diet on adults and found myostatin null mice were more susceptible to diet-induced obesity through a mechanism involving impairment of inter-organ fat utilization.

**Conclusions:**

Loss of myostatin alters fatty acid uptake and oxidation in skeletal muscle and liver. We show that abnormally high metabolic activity of fat in myostatin null mice is decreased by a high-fat diet resulting in excessive adipose deposition and lipotoxicity. Collectively, our genetic loss-of-function studies offer an explanation of the lean phenotype displayed by a host of animals lacking myostatin signalling.

**Electronic supplementary material:**

The online version of this article (doi:10.1186/s13395-015-0063-5) contains supplementary material, which is available to authorized users.

## Background

A chronic imbalance between dietary intake and energy expenditure results in an accumulation of adipose tissue and subsequent development of obesity. Given the global prevalence of obesity and metabolic/cardiovascular disorders, a better understanding of the fundamental principles which govern diet-induced metabolic pathophysiology is requisite to advance novel anti-obesity therapies [1].

Obesity affects not only the adult but, in pregnant women, the development of the foetus. Irrefutable evidence exists showing that abnormal intrauterine environment increases the susceptibility of the offspring to a host of diseases including osteoporosis, high blood pressure, insulin resistance, type 2 diabetes and even cancer [2–8]. The lifelong effects of exposure to a high-fat diet during pregnancy establishes a vicious cycle in that large babies have increased probability of being obese and therefore as adults will give birth to overweight children.

Recent evidence suggests loss of skeletal muscle metabolic plasticity is central in the development of obesity and metabolic disease. This is highlighted by numerous studies, from mouse to man, implicating the role of skeletal muscle fibre type composition, size, oxidative enzyme activity and lipid content as causal factors for predicting or predisposing to obesity [9–15]. A reduction in the oxidative capacity of skeletal muscle to uptake and utilize circulating lipids along with attenuated oxidative enzymatic activity, increases muscle lipid content and smaller fibre size are contributing factors in the aetiology of obesity [9, 12]. Conversely, increased fatty acid oxidation in peripheral tissues such as skeletal muscle and adipose tissue is protective against fat accumulation in adipose tissue and obesity [16]. Targeting of myostatin (Mstn) activity or signalling has emerged as a potential strategy to combat obesity as deletion of Mstn is accompanied by a hypermuscular phenotype. Muscle hypertrophy is accompanied by a change in the metabolic profile of the tissues signified by a huge increase in the number of glycolytic fibres and deficit in mitochondrial number. With regards to adiposity, it has been shown that loss of myostatin in leptin-deficient mice is followed by reduced accumulation of whole-body fat content [17]. In addition, transgenic expression of the myostatin propeptide, a molecule that maintains myostatin in an inactive form, is proposed to be protective against high-fat diet-induced obesity [18]. Recent reports on Mstn knock out (Mstn^−/−^) mice or treatment with myostatin antagonists (e.g. soluble activin type IIB receptor) showed resistance to develop obesity in response to high-fat diet (e.g. [16]). Paradoxically, a huge body of evidence shows that an oxidative muscle profile, rather than glycolytic protects against obesity (e.g. [19, 20]).

We hypothesised that myostatin inhibition should protect not only the mother but also its developing foetus from the detrimental effects of a high-fat diet. Contrary to our expectations, we found that gestational high-fat diet had detrimental effects on skeletal muscle development by impairing muscle fibre formation. Furthermore, we provide evidence that Mstn deletion is not beneficial in adult mice subjected to high-fat diet. We carried out an analysis of the three major fat handling tissues in the body to develop a mechanistic explanation for our findings. Detailed quantitative gene expression analysis revealed that the oxidative profile is attenuated in muscle. Our data demonstrates that a high-fat diet induces abnormal fatty acid uptake and oxidation programmes in the skeletal muscle and liver of myostatin deficient mice. Finally, we provide evidence that a high-fat diet induced abnormal programmes of fat oxidation and energy dissipation specifically in Mstn^−/−^ mice. We suggest that a culmination of the abnormal responses of muscle, liver and adipose tissues results in excessive fat deposition in Mstn^−/−^ mice.

## Methods

### Ethical approval

All research conducted on animals was performed under a project license from the United Kingdom Home Office in agreement with the Animals (Scientific Procedures) Act 1986. All procedures were approved by the University of Reading Animal Care and Ethical Review Committee. Animals were humanely sacrificed via Schedule 1 killing between 0800–1300.

### Animal maintenance

Healthy C57Bl/6 (WT) and Mstn^−/−^ mice were bred and maintained according to the NIH Guide for Care and Use of Laboratory animals, approved by the University of Reading in the biological resource unit of Reading University whereby they were housed under standard environmental conditions (20–22 °C, 12–12 h light–dark cycle) and provided food and water ad libitum. All mice were of 4–5 months of age at the commencement of the study. Experimental groups were composed of 5–9 mice each. Mstn^−/−^ mice were a gift of Se-Jin Lee (Johns Hopkins USA).

### High-fat diet protocol

Mice were caged individually and were randomly subjected to a purified laboratory high-fat diet (HF diet) regime or supplied with a standard laboratory mouse chow. High-fat diet was obtained from special diet services (SDS) with 45, 20 and 35 % of total energy intake deriving from fat, protein and carbohydrates, respectively (diet code: 824053). Animals were monitored daily and maintained under high-fat diet conditions for a maximum of 10 weeks. Upon completion of the study the heart, extensor digitorum longus (EDL) plantaris, tibialis anterior, gastrocnemius, vastus lateralis, soleus and rectus femoris muscles as well as the liver and white adipose tissue (WAT) from the retroperitoneal visceral fat pad from male and female mice were dissected and weighed. Embryos were obtained at embryonic stage E18.5 from timed pregnant female mice being on a high fat diet for 9–10 weeks at the time of tissue harvesting. Embryo hind-limbs were embedded in Tissue Tech freezing medium (Jung) cooled by dry ice/ethanol. Immunocytochemistry was performed on serial cryosections as described previously [21]. No major sex specific difference was found between the two genotypes and most of the results presented are from male mice as these were the bigger cohorts.

### Clinical chemistry analysis of blood

Blood was collected by heart puncture in presence of lithium heparin anticoagulant and plasma separated by centrifugation. Up to 200 μl of plasma were analysed with a Beckman Coulter AU680 clinical chemistry analyser.

### Histological analysis and immunohistochemistry

Following dissection, muscle was immediately frozen in liquid nitrogen-cooled isopentane and mounted in Tissue Tech freezing medium (Jung) cooled by dry ice/ethanol. Immunohistochemistry was performed on 10 μm cryosections which were dried for 30 min before the application of block wash buffer (PBS with 5 % foetal calf serum (*v*/*v*), 0.05 % Triton X-100). Antibodies were diluted in wash buffer 30 min before use. Myosin heavy chain (MHC) type I, IIA and IIB isoforms were identified by using A4.840 IgM (1:1 dilution), A4.74 IgG (1:4 dilution) and BF-F3 IgM (1:1 dilution) supernatant monoclonal primary antibodies (Developmental Studies Hybridoma Bank). An IgG rabbit polyclonal antibody against laminin (Sigma) was used at a concentration of 1:300. Phospho-NF-κB antibody staining (Ser536) was performed at a dilution of 1:200 (93H1 Cell Signalling UK). Macrophage marker F4/80 (1:200, CA497R, AbD Serotec) was histologically visualised by using the Vectastain Elite ABC kit (Vector Labs, UK).

Primary antibodies were detected using Alexa Fluor 488 goat anti-mouse IgG (Molecular Probes A11029, 1:200), Alexa Fluor 633 goat anti-mouse IgM (Molecular Probes A21046, 1:200) and Alexa Fluor 488 goat-anti-rabbit IgG (Molecular Probes A11008, 1:300) secondary antibodies.

### Succinate dehydrogenase (SDH) staining

Transverse EDL muscle sections were incubated for 3 min at room temperature in a sodium phosphate buffer containing 75 mM sodium succinate (Sigma), 1.1 mM nitroblue tetrazolium (Sigma) and 1.03 mM phenazine methosulphate (Sigma). Samples were then fixed in 10 % formal-calcium and cleared in xylene prior to mounting with DPX mounting medium (Fisher). Photographic quantification of the samples was performed on a Zeiss Axioskop2 microscope mounted with an Axiocam HRc camera. Axiovision Rel. 4.8 software was used to capture the images.

### Oil Red O staining

10 μm thick liver cryosections were washed in PBS and rinsed in 60 % isopropanol. Sections from all experimental groups were processed simultaneously with freshly prepared Oil Red O working solution for 15 min. Nuclei were counterstained with alum hematoxylin for 1 min. Sections were mounted in aqueous mounting medium and images were captured with a bright-field microscope.

### Quantitative PCR

Tissue samples were disrupted/homogenised in PureZOL™ (Biorad) in a Tissue-lyzer (Qiagen) using stainless steel beads (30 Hz for a total of 4 min). Total RNA was isolated using the Aurum™ (Biorad) RNA isolation kit according to manufacturer’s directions. For QPCR, total RNA was deoxyribonuclease-treated on-column and transcribed to complementary DNA using iScript™ (Biorad) following manufacturer’s protocol. QPCR was performed with the TaqMan method (using the Roche Universal Probe Library System) on an ABI StepOnePlus Real-Time PCR System. Relative expression was calculated using the ΔΔ*C*_t_ method with normalisation to the housekeeping gene cyclophilin-B. Specific primer/probe sequences are available on request.

### Muscle tension measurements

Dissection of the hind limb was carried out under oxygenated Krebs solution (95 % O_2_ and 5 % CO_2_). Under circulating oxygenated Krebs solution one end of a silk suture was attached to the distal tendon of the EDL and the other to a Grass Telefactor force transducer (FT03). The proximal tendon remained attached to the tibial bone. The leg was pinned to a Sylgard-coated experimental chamber. Two silver electrodes were positioned longitudinally on either side of the EDL. A constant voltage stimulator (S48, Grass Telefactor) was used to directly stimulate the EDL which was stretched to attain the optimal muscle length to produced maximum twitch tension (*P*_t_). Tetanic contractions were provoked by stimulus trains of 500 ms duration at, 10, 20, 50, 100 and 200 Hz. The maximum tetanic tension (*P*_o_) was determined from the plateau of the frequency-tension curve. Specific force was estimated by normalising tetanic force to EDL muscle mass (g).

### Exercise fatigue test

Following completion of a 6-week dietary intervention, mice were acclimatised in three sessions to running on a treadmill (10 m/min for 15 min followed by a 1 m/min increase per minute to a maximum of 12 m/min) (Columbus Instruments Model Exer 3/6 Treadmill, Serial S/N 120416). Exhaustion was determined by exercising the mice at 12 m/min for 5 min, followed by 1 m/min increases to a maximum of 20 m/min until the mouse was unable to run.

### ^1^H NMR spectroscopy-based metabonomic analysis

Polar metabolites were extracted from gastrocnemius muscle using the protocol previously described by Beckonert et al. [22]. Briefly, 40–50 mg of muscle tissue was snap frozen in liquid nitrogen and finely ground in 300 μL of chloroform:methanol (2:1) using a tissue lyzer. The homogenate was combined with 300 μL of water, vortexed and spun (13,000*g* for 10 min) to separate the aqueous (upper) and organic (lower) phases. A vacuum concentrator (SpeedVac) was used to remove the water and methanol from the aqueous phase before reconstitution in 550 μL of phosphate buffer (pH 7.4) in 100 % D_2_O containing 1 mM of the internal standard, 3-(trimethylsilyl)-[2,2,3,3,-^2^H_4_]-propionic acid (TSP). For each sample, a standard one-dimensional nuclear magnetic resonance (NMR) spectrum was acquired with water peak suppression using a standard pulse sequence (recycle delay (RD)-90°-*t*_1_-90°-*t*_m_-90°-acquire free induction decay (FID)). RD was set as 2 s, the 90° pulse length was 16.98 μs, and the mixing time (*t*_m_) was 10 ms. For each spectrum, eight dummy scans were followed by 128 scans with an acquisition time per scan of 3.8 s and collected in 64 K data points with a spectral width of 12.001 ppm. ^1^H NMR spectra were manually corrected for phase and baseline distortions and referenced to the TSP singlet at δ 0.0. Spectra were digitised using an in-house MATLAB (version R2009b, the Mathworks, Inc.; Natwick, MA) script. To minimize baseline distortions arising from imperfect water saturation, the region containing the water resonance was excised from the spectra. Principal components analysis (PCA) was performed with Pareto scaling in MATLAB using scripts provided by Korrigan Sciences Ltd, UK.

### Lipid profiling

Cellular extracts from gastrocnemius were quantified on a time-of-flight mass spectrometer (micrOTOF Q II from Bruker Daltonics, Germany) equipped with an ESI standard sprayer (Apollo II ESI source) according to previously published methods [23–26]. Samples were injected using an autosampler “ultimate WPS-3000TSL” and a multi-channel pump “ultimate 3400 M” (Thermo Fisher, Germany). Lipids were extracted according to [24]. Dried samples were reconstituted in 250 μl MS-mix buffer [chloroform/methanol/ammonium formiate (2/1/0.1 %)] and 10 μl were infused to an Ascentis Express C8 analytical column (Sigma-Aldrich, Germany) at a flow rate of 260 μl/min. Chromatography was performed using a multistep gradient with buffer A (acetonitril/water 60/40) and buffer B (2-propanol/water 97/3) containing 0.1 % ammonium formiate, starting with A/B 68/32 and ending A/B 3/97 after 30 min. The mass spectrometry data were processed with Target Analysis Software (version 1.2) from Bruker Daltonics. Sample data were processed for eight lipid subclasses: triglycerides, sphingomyelins, phosphatidylserines, phosphatidylethanolamines, phosphatidylcholines, lyso-phosphatidylcholines, cholesterolesters and ceramides, using internal standards (TAG 19:0/19:0/19:0; PC 17:0/17:0; LPC 17:0; SM 17:0; PE 17:0; CE 17:0; CM 17:0; SM 17:0).

### Statistical analysis

Data are presented as mean ± SD. Significant differences between two groups were performed by Student’s *t* test for independent variables. Differences among groups were analysed by two-way analysis of variance (ANOVA; genotype x diet) followed by Bonferroni’s multiple comparison tests. Differences were considered statistically significant at *p* < 0.05.

## Results

### Effect of maternal high-fat diet on embryonic muscle development

**Fig. 1:**
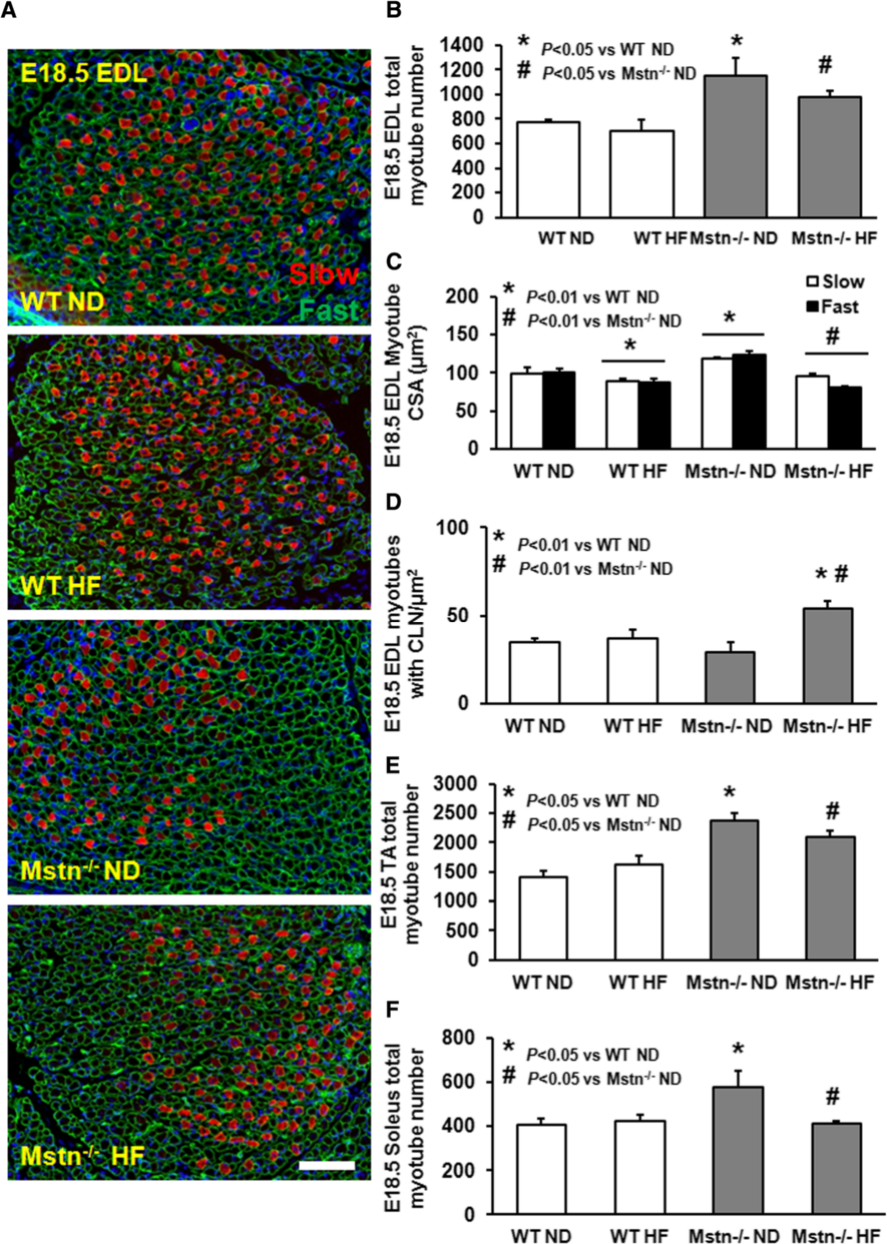
Effect of high fat on embryonic muscle development. EDL muscle morphology at developmental stage E18.5 from wild type (WT) and myostatin null (Mstn^−/−^) embryos from mice subjected to maternal normal (ND) or a high-fat (HF) diet. **a** Representative immunofluorescence images depicting primary (slow; *red*) and secondary (fast; *green outline*) myotubes from EDL. Scale 100 μm. **b** Total myotube number in EDL muscle at E18.5. **c** Myotube cross-sectional area and **d** myotubes with central nuclei per unit area. **e**, **f** Total myotube number of the TA and soleus muscles at E18.5 from WT and Mstn^−/−^ embryos from mothers under a ND or HF diet. ANOVA; (*) *P* < 0.05 vs. WT ND; (^#^) *P* < 0.05 vs. Mstn^−/−^ ND. Data are from *N* = 6–8 embryos per group

**Fig. 2:**
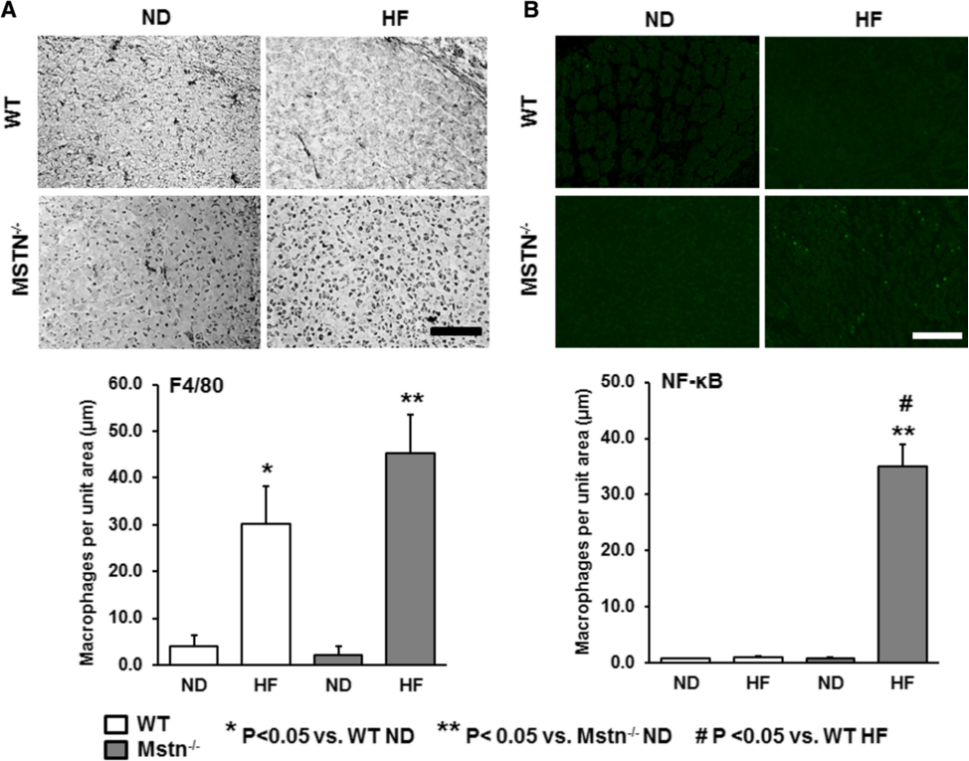
Effect of high-fat diet on inflammatory markers of embryonic muscle. **a** Evidence of intramuscular macrophages stained for F4/80 and **b** NF-κB immunofluorescence in embryonic muscle. Scale 100 μm, ANOVA; (*) *P* < 0.05 vs. WT ND; (**) *P* < 0.05 vs. Mstn^−/−^ ND; (^#^) *P* < 0.05 vs. WT HF. Data are from *N* = 5 embryos per group

Attenuation of myostatin signalling has been reported to increase fatty acid metabolism. Here, we investigated the effect of high fat on muscle development on a genetic background that lacks myostatin (Mstn^−/−^); (Fig. 1a–f). Muscle morphology from littermate embryos was performed at the developmental stage E18.5, when secondary myogenesis is considered to be approaching postnatal levels. As reported previously [21], total myotube number of the EDL was significantly higher in the Mstn^−/−^ embryos compared to the WT ones. However, we found a significantly compromised myotube number of 15 % in the EDL of Mstn^−/−^ embryos from mothers raised on a HF diet (Fig. 1a, b). Concordantly, a 30 and 12 % reduction in total myotube number was evident in the soleus and TA muscles, respectively, of Mstn^−/−^ embryos from mothers on a HF diet in the absence of any differences in WT embryos (Fig. 1e,f). EDL primary and secondary myotube cross-sectional area (CSA) was significantly reduced by 10 % in WT embryos from mothers kept on a HF diet. Most importantly, a significant interaction between genotype and diet revealed a significantly higher CSA reduction in both primary (slow) and in particular secondary (fast) EDL myotubes of Mstn^−/−^ embryos by 20 and 35 %, respectively (Fig. 1c). In addition, the number of myotubes with centrally located nuclei, as an indication of myotube remodelling and regeneration were significantly higher in the Mstn^−/−^ HF mice (Fig. 1d). We examined the level of inflammation as a means to possibly explain the decrease in muscle development. Histological staining for F4/80, a macrophage marker revealed a significant increase for both genotypes in response to high-fat diet (Fig. 2a). We also noticed a significant increase in activated NF-κB immunostaining only in the Mstn^−/−^ HF embryos (Fig. 2b). Taken together, these novel findings suggest that maternal subjection to HF diet has more severe and detrimental impact on muscle development on Mstn^−/−^ compared to WT embryos possibly through an induction of an inflammatory response.

### Effect of high-fat diet on mouse gross anatomy

**Fig. 3:**
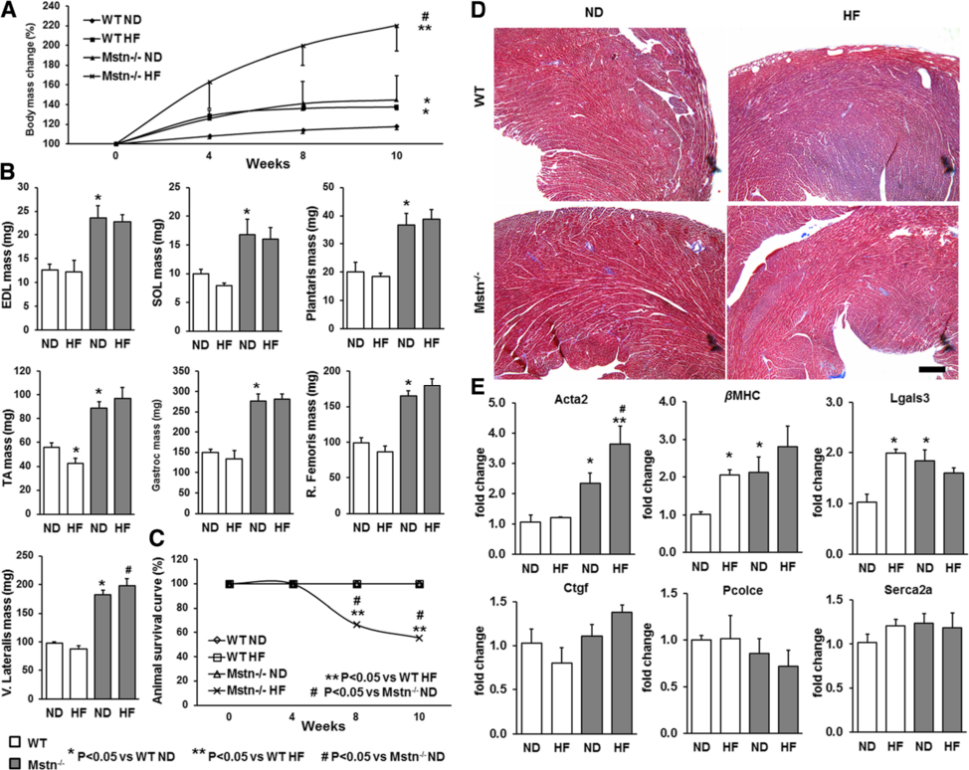
Effect of high-fat diet on body mass, skeletal muscle masses, animal survival curve and cardiac muscle. **a** Percent changes of body mass in wild type (WT) and myostatin null (Mstn^−/−^) mice subjected to either a normal (ND) or a high-fat (HF) diet for 10 weeks. ANOVA; (*) *P* < 0.05 vs. WT ND; (^#^) *P* < 0.05 vs. WT HF; (**) *P* < 0.005 vs. Mstn^−/−^ ND. **b** Effect of high-fat (HF) diet on EDL, soleus, plantaris, tibialis anterior (TA), gastrocnemius, rectus femoris and vastus lateralis muscle masses. **c** Animal survival curve in wild type (WT) and myostatin null (Mstn^−/−^) mice subjected to either a normal (ND) or a high-fat (HF) diet. ANOVA; (*) *P* < 0.05 vs. WT normal diet (ND); (^#^) *P* < 0.05 vs. Mstn^−/−^ ND. A 33 and 45 % reduction of animal survival in Mstn^−/−^ HF diet cohort was observed at the 8-week and 10-week time point, respectively. **d** Trichrome staining on the heart muscle. Scale 100 μm. **e** mRNA levels of key markers involved in cardiac hypertrophy and fibrosis. ANOVA; (*) *P* < 0.05 vs. WT ND, (#) P < 0.05 vs. WT HF; (**) P < 0.005 vs. Mstn−/− ND ; *N* = 5 male mice per group

The above findings demonstrated that a maternal high-fat diet had a detrimental effect on the foetal muscle development programme. We also noted that pregnant as well as virgin male and female Mstn^−/−^ mice raised on a high-fat diet developed precocious levels of visceral fat. The effect of high-fat diet on percent body mass revealed a significant increase in WT mice by 20–22 % that was evident throughout the study (Fig. 3a). Surprisingly, Mstn^−/−^ mice (irrespective of sex) subjected to a high-fat diet for 10 weeks elicited a 37–76 % increase in body mass. Moreover, Mstn^−/−^ mice are known for their hypermuscular phenotype compared to wild-type littermates and individual hind limb muscles were heavier compared to wild-type cohorts. Curiously, high-fat diet did not affect individual muscle masses in either genotype, with the exception of TA and vastus lateralis in WT and Mstn^−/−^, respectively (Fig. 3b). Importantly, high-fat diet compromised Mstn^−/−^ mice survival curves by 33–45 % in male (Fig. 3c) and female mice (data not shown). These phenotypic findings indicate that HF diet has deleterious effects on body mass changes and life span in the Mstn^−/−^ mice. Histological examination of heart did not reveal any fibrotic lesions (Fig. 3d). We furthermore looked for transcript levels of key factors playing a role in pathological heart hypertrophy and fibrosis as a possible explanation for mortality (i.e. smooth muscle α actin*, Acta2*; β myosin heavy chain, *βMHC*; lectin, galactoside-binding soluble 3, *Lgals3*; connective tissue growth factor, *Ctgf*; procollagen C-endopeptidase enhancer, *Pcolce*; and sarcoplasmic reticulum Ca^2+^ ATPase, *Serca2a*). *Acta2* mRNA were elevated in Mstn^−/−^ mice compared to WT and were further increased in Mstn^−/−^ mice in response to HF diet (Fig. 3e). Mstn^−/−^ ND mice showed significantly higher mRNA levels for *βMHC* and *Lgals3* compared to WT ND and high-fat diet led to increased levels for both genes only in WT HF mice. No changes were found for *Ctgf, Pcolce and Serca2a*.

### Effect of high-fat diet on blood lipids, liver function markers and cellular damage markers

**Fig. 4:**
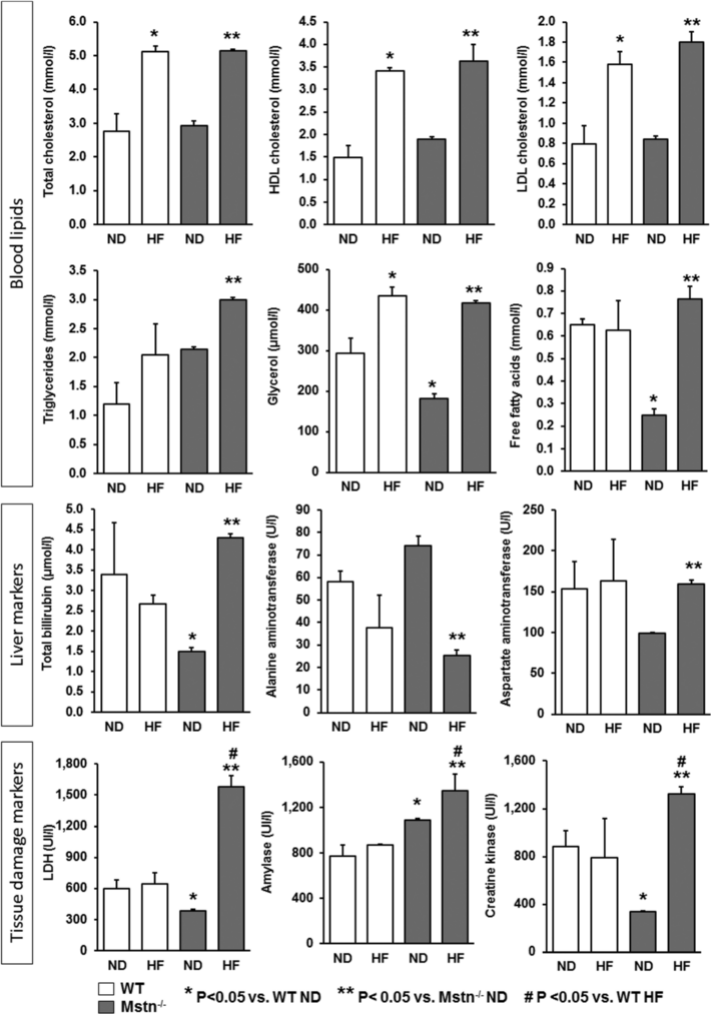
Effect of high-fat diet on blood lipids, liver function markers and markers of cellular damage. ANOVA; (*) *P* < 0.05 vs. WT ND; (**) *P* < 0.05 vs. Mstn^−/−^ ND; (^#^) *P* < 0.05 vs. WT HF. Data are from *N* = 5 male mice per group

Plasma lipids were profiled with a Beckman Coulter AU680 clinical chemistry analyser. High-fat diet led to a significant increase of total-HDL and LDL-cholesterol and glycerol in both WT and Mstn^−/−^ mice of either sex (Fig. 4). Triglyceride levels were twice the levels in Mstn^−/−^ mice on a normal diet compared to similarly fed WT animals. In addition, triglycerides and free fatty acids were significantly increased only in the Mstn^−/−^ HF mice. Liver function markers were unaffected in WT HF mice, while Mstn^−/−^ HF mice showed an increase for bilirubin and aspartate aminotransferase as well as a decrease for alanine aminotransferase, respectively (Fig. 4). Interestingly, markers of cellular damage (i.e. lactate dehydrogenase, amylase and creatine kinase) were significantly increased only in the Mstn^−/−^ HF mice (Fig. 4) and Additional file 1: Table S1.

### Effect of high-fat diet on muscle metabolic properties

**Fig. 5:**
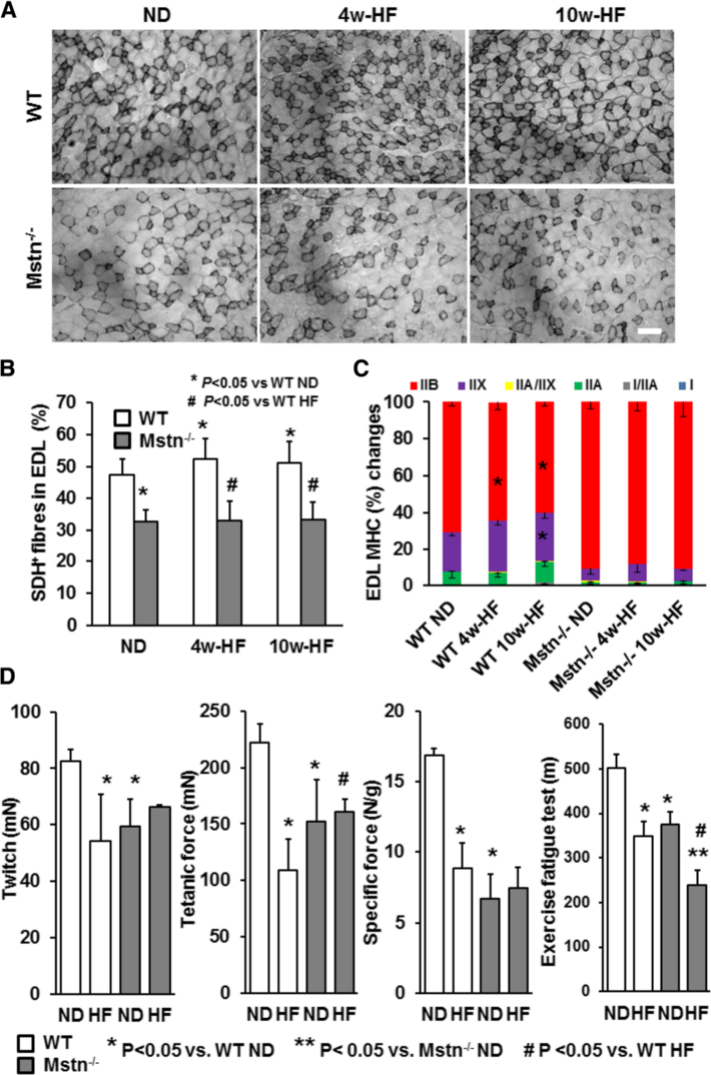
Effect of high-fat diet on EDL muscle mitochondrial activity, fibre type and contractile properties. **a** EDL succinate dehydrogenase (SDH) activity in wild type (WT) and myostatin null (Mstn^−/−^) mice subjected to a normal (ND) or high-fat (HF) diet for 4 and 10 weeks. Representative histochemical staining for SDH. Scale 100 μm. *N* = 6 mice per group. **b** Quantification of SDH-positive (SDH+) fibres among groups. ANOVA; (*) *P* < 0.05 vs. WT ND; (^#^) *P* < 0.05 vs. WT HF. **c** Fibre type changes on EDL from wild type (WT) and myostatin null (Mstn^−/−^) mice subjected to a normal (ND) or high-fat (HF) diet for 4 and 10 weeks. ANOVA; (*) *P* < 0.05 vs. WT ND; (^#^) *P* < 0.05 vs. WT HF. *N* = 6 mice per group. **d** EDL muscle contractile properties in wild type (WT) and myostatin null (Mstn^−/−^) mice subjected to a normal (ND) or high-fat (HF) diet. ANOVA; (*) *P* < 0.05 vs. WT ND; (^#^) *P* < 0.05 vs. WT HF. *N* = 6 mice per group. Exercise fatigue test of from wild type (WT) and myostatin null (Mstn^−/−^) mice subjected to a normal (ND) or high-fat (HF) diet for 6 weeks. ANOVA; (*) *P* < 0.05 vs. WT ND; (^#^) *P* < 0.05 vs. Mstn^−/−^ ND; (**) P < 0.05 vs. WT HF. *N* = 5 male mice per group

Fat catabolism takes place in the mitochondria. Given the impaired mitochondrial contents that characterise the muscles of Mstn^−/−^ mice [27], we next focused our analysis on mitochondrial respiration in skeletal muscle from WT and Mstn^−/−^ mice in response to high-fat diet. Mitochondrial activity estimated via SDH histological staining showed a significant increase in SDH positive fibres in WT mice after both 4 and 10 weeks of HF diet by 5 % (Fig. 5a, b). As expected, Mstn^−/−^ mice had fewer SDH positive fibres compared to WT cohorts but subjection to high-fat diet did not affect SDH levels in Mstn^−/−^ mice. This finding may be taken as functional compensation in the WT mice to metabolise the excess of fat supplied by nutrition or alternatively as failure to appropriately augment mitochondrial activity in response to high-fat diet by the Mstn^−/−^ mice. We next analysed muscle fibre composition of EDL muscle in order to decipher the role of skeletal muscle morphology on total body metabolism and the development of obesity.

In line, with the SDH findings, we observed a fibre type shift from glycolytic IIB to more oxidative types (i.e. IIX and IIA) after 4 and 10 weeks of HF diet in the wild-type mice. In contrast, no significant changes were found in EDL muscle fibre types for Mstn^−/−^ mice subjected to HF diet (Fig. 5c). Taken together, the changes in SDH and MHC isoforms found in WT mice potentially indicate functional metabolic alterations that favour fat buffering and utilization in a system with excess fat content. However, this mechanism is not activated robustly in Mstn^−/−^ animals.

### Effect of high-fat diet on muscle contractile properties

We next determined the effect of HF diet on EDL muscle contractile properties. As previously reported by us and others, EDL tetanic and specific force were significantly lower in Mstn^−/−^ EDL muscles compared to WT mice subjected to normal diet [28, 29]. Twitch tension revealed a significant interaction between diet and genotype originating mainly in a 30 % reduction for WT mice in response to HF diet (Fig. 5d). We also observed a significant reduction in tetanic tension for WT mice on HF diet, which exceeded the known low levels found in Mstn^−/−^ mice (Fig. 5d). By normalising tetanic force to wet muscle mass, we found a sharp reduction in specific tension for WT mice reaching the known attenuated levels of Mstn^−/−^ mice (Fig. 5d). Overall, HF diet did not have any impact on the contractile properties of Mstn^−/−^ mice. These findings indicate that despite the metabolic remodelling of EDL muscle for WT mice, HF has detrimental effects on muscle contractile properties. In addition, the already compromised contractile properties of Mstn^−/−^ EDL muscle were not further affected by HF diet. When animals were challenged by an exercise fatigue protocol, we noticed an attenuated exercise tolerance for both genotypes under HF diet which was more pronounced in the Mstn^−/−^ mice (Fig. 5d).

### Effect of high-fat diet on the expression of genes controlling metabolic activity in skeletal muscle

**Fig. 6:**
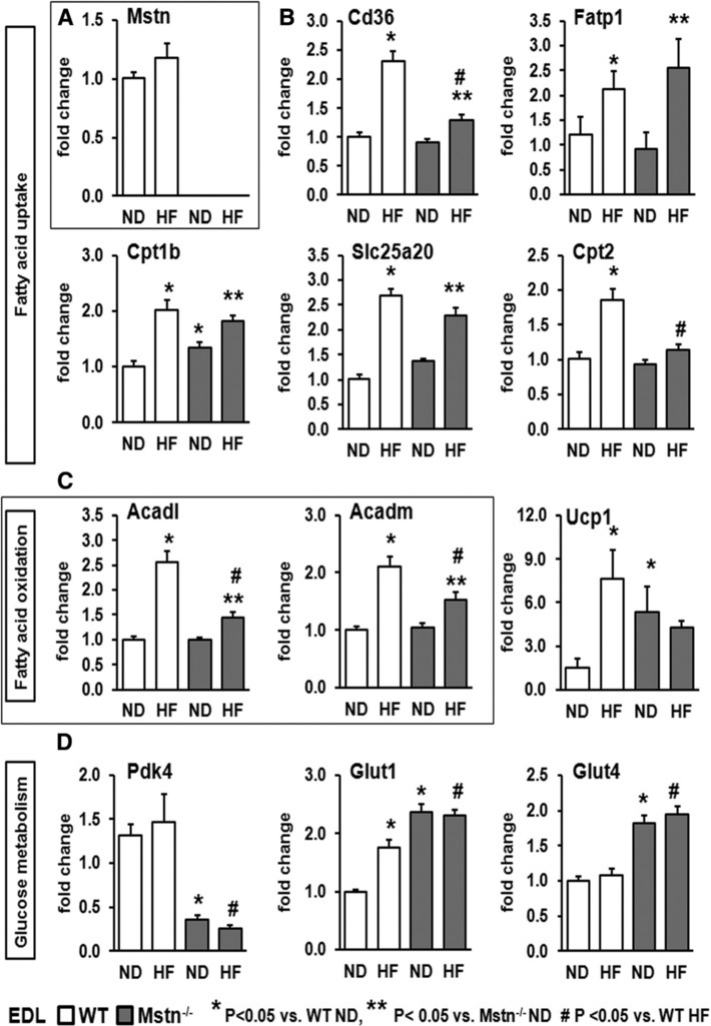
Effect of high-fat diet on EDL muscle gene expression. EDL gene expression levels of **a** *Myostatin*, **b** key factors regulating fatty acid uptake (i.e. *Cd36, Fatp1, Cpt1b, Slc25a20* and *Cpt2*), **c** fatty acid oxidation (i.e. *Acadl* and *Acadm*) as well as U*ucp1* and **d** glucose metabolism (i.e. *Pdk4, Glut1 and Glut 4*). ANOVA; (*) *P* < 0.05 vs. WT ND; (^#^) *P* < 0.05 vs. WT HF; (**) *P* < 0.05 vs. Mstn^−/−^ ND. *N* = 6 male mice per group

We next determined the gene expression patterns of *Mstn* and key metabolic regulators in EDL. HF diet did not affect EDL Mstn mRNA levels in the WT mice and as expected Mstn transcript was not detectable in the Mstn^−/−^ mice (Fig. 6a). We determined the transcript levels of key genes involved in fatty acid uptake (i.e. *Cd36*, *Fatp1*, *Cpt1b*, *Slc25a20* and *Cpt2*), mitochondrial fatty acid oxidation (i.e. *Acadl* and *Acadm*) as well as glucose metabolism (i.e. *Pdk4*, *Glut1* and *Glut 4*; Fig. 6b–d). High-fat diet resulted in a significant induction of gene products that regulate fatty acid uptake (i.e. *Fatp1*, *Cpt1b* and *Slc25a20*) in EDL muscle in both WT and Mstn^−/−^ mice (Fig. 6b). However, a significant interaction was evident between diet and genotype originating in a more robust induction of mRNA levels in the WT mice compared to Mstn^−/−^ with regard to fatty acid uptake genes *Cd36* (i.e. 1.3 in WT vs. 0.3-fold change in Mstn^−/−^) and *Cpt2* (i.e. 0.9-fold in WT vs. 0.2-fold change in Mstn^−/−^) as well as the fatty acid oxidation genes *Acadl* (i.e. 1.6-fold in WT vs. 0.5-fold change in Mstn^−/−^) and *Acadm* (i.e. 1.1-fold in WT vs. 0.4-fold change in Mstn^−/−^; Fig. 6b, c). A significant main effect of genotype was apparent on mRNA levels of genes that regulate glucose metabolism (i.e. *Pdk4*, *Glut1* and *Glut4*), due to the predominant glycolytic muscle phenotype of the Mstn^−/−^ mice. Moreover, HF diet did not affect expression of glucose metabolism genes with the exception of a significant increase on *Glut1* (constitutive glucose transporter in the fasting state [30]) levels only in the WT cohort (Fig. 6d). We also found that wild-type mice subjected to HF diet show a 4-fold upregulation of *ucp1* in the EDL muscle, which is blunted in the Mstn^−/−^ HF diet mice (Fig. 6c). A similar profile of gene expression was discovered when we examined transcripts in the soleus muscle (Additional file 2: Figure S1). Collectively, these data show a sub-optimal transcriptional adaptation of muscle HF in the absence of myostatin.

### Effect of high-fat diet on the expression of genes controlling metabolic activity in liver

**Fig. 7:**
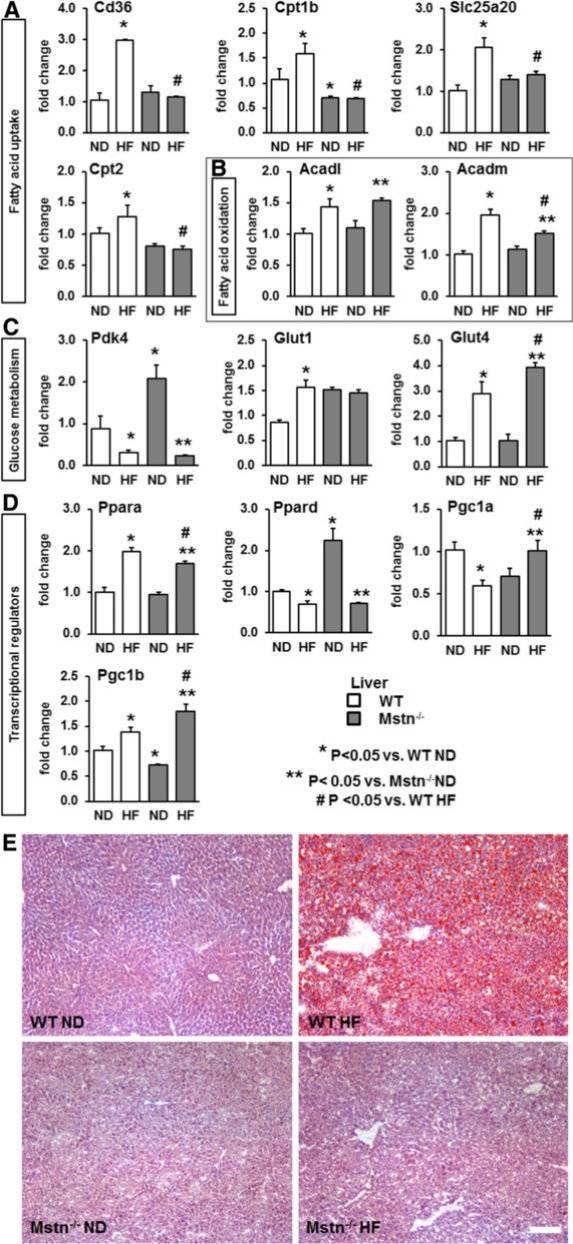
Effect of high-fat diet on liver gene expression and liver fat deposition. Liver gene expression levels of **a** key factors regulating fatty acid uptake (i.e. *Cd36, Cpt1b, Slc25a20 and Cpt2*), **b** fatty acid oxidation (i.e. *Acadl* and *Acadm*), **c** glucose metabolism (i.e. *Pdk4, Glut1 and Glut 4*) as well as **d** transcriptional regulators (i.e. *Ppara, Ppard, Pgc1a* and *Pgc1b*). ANOVA; (*) *P* < 0.05 vs. WT ND; (^#^) *P* < 0.05 vs. WT HF; (**) *P* < 0.05 vs. Mstn^−/−^ ND. *N* = 6 mice per group. **e** Representative histological images of liver fat contents by Oil Red O staining from wild type (WT) and myostatin null (Mstn^−/−^) male mice subjected to a normal (ND) or high-fat (HF) diet for 10 weeks. Scale 100 μm

As the EDL of Mstn^−/−^ mice showed a blunted response to HF, we next determined the gene expression patterns of key metabolic regulators in the liver, another major site regulating adiposity. We measured mRNA levels of genes that regulate fatty acid uptake (i.e. *Cd36*, *Cpt1b*, *Slc25a20* and *Cpt2*), fatty acid oxidation (i.e. *Acadl* and *Acadm*), and glucose metabolism (i.e. *Pdk4*, *Glut1* and *Glut4*) as well as transcriptional regulators of energy metabolism from the family nuclear receptors (i.e. *Ppara*, *Ppard*, *Pgc1a* and *Pgc1b*). We found that HF diet induced genes involved in fatty acid uptake and oxidation only in the WT cohort (Fig. 7a–d). Conversely, gene expression level changes were blunted in the liver of Mstn^−/−^ HF mice for all genes regulating fatty acid uptake, except *Acadl* and *Acadm* (involved in fatty acid oxidation). With regard to genes that control glucose metabolism, HF diet increased mRNA levels of glucose transporters *Glut1* and *Glut4* and decreased *Pdk4* levels in the WT mice, all changes suggesting an increased metabolic response and glucose utilization (Fig. 7c). HF diet significantly reduced *Pdk4*, did not affect *Glut1* and increased *Glut4* mRNA levels in the Mstn^−/−^ mice (Fig. 7c). With the exception of *Glut1*, the changes on mRNA levels of *Pdk4* and *Glut4* also denote increased glucose metabolism and transport within the liver. We also examined the expression of *Ppara*, a master regulator of lipid metabolism in the liver and adipose tissue [31]. *Ppara* was upregulated in WT mice in response to a high-fat diet. However, the gene was induced less robustly at significant levels by high fat in gene Mstn^−/−^ mice (Fig. 7d). Taken together, this data indicates that the transcriptional regulation of fat metabolism by the liver in Mstn^−/−^ mice is compromised. This finding suggests that Mstn^−/−^ mice do not handle the excess of dietary fats like WT mice. We therefore conducted a profiling of tissue fat content for fat droplet deposition in the liver (Fig. 7e). Oil Red O staining revealed a pronounced fat accumulation in the liver of WT mice maintained on a HF diet, a profile that differed greatly from the control condition. Significantly and in contrast to WT mice, we found that there was no fat deposition in the livers of Mstn^−/−^ mice raised on a high-fat diet. Indeed, there was no change in Oil Red profiles between Mstn^−/−^ mice raised on HF compared to normal diet.

### Effect of high-fat diet on gene expression patterns of white fat

**Fig. 8:**
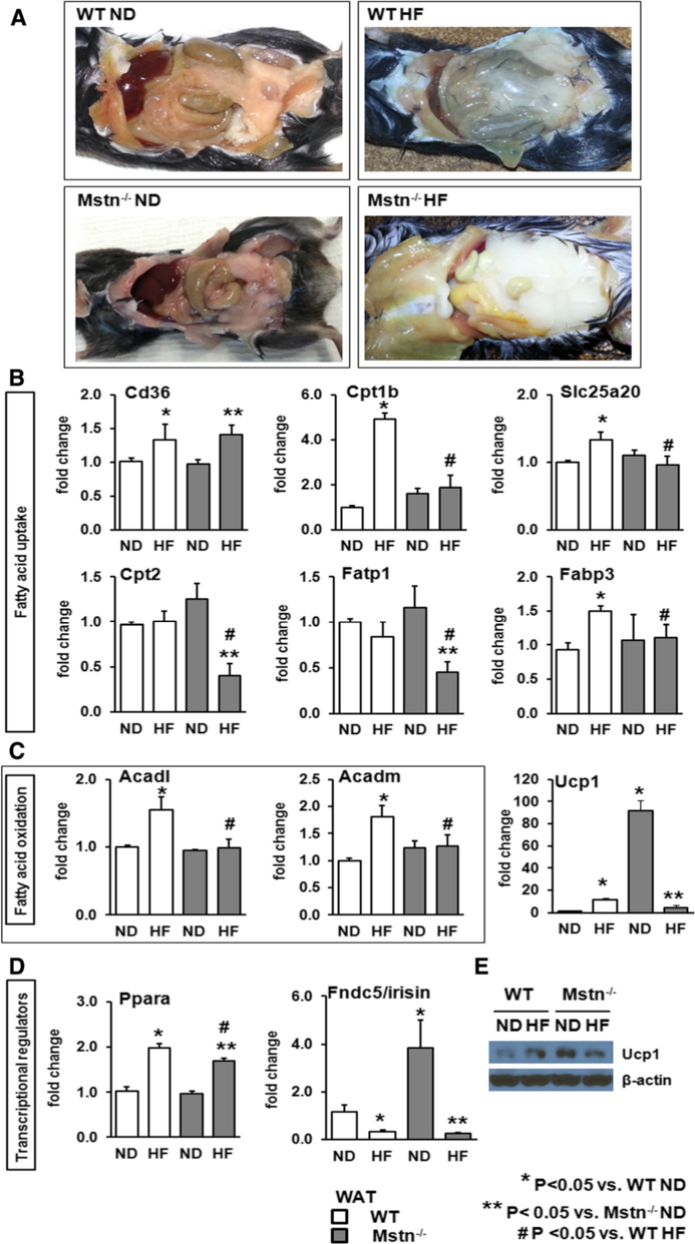
Effect of high-fat diet on body fat contents and on white adipose tissue gene expression. **a** Visceral fat deposition in WT and Mstn^−/−^ mice subjected to either a ND or a HF diet. Note the excessive fat contents in the Mstn^−/−^ HF cohort. White adipose tissue (WAT) gene expression levels of **b** key factors regulating fatty acid uptake (i.e. *Cd36, Cpt1b, Slc25a20, Cpt2, Fatp1* and *Fabp3*), **c** fatty acid oxidation (i.e. *Acadl* and *Acadm*) and *Ucp1*. **d** Fatty acid transcriptional regulator *Ppara* and regulator of Upc1-*Fndc5*/*irisin*. ANOVA; (*) *P* < 0.05 vs. WT ND; (^#^) *P* < 0.05 vs. WT HF; (**) *P* < 0.05 vs. Mstn^−/−^ ND. *N* = 6 mice per group. **e** Representative immunoblot for ucp1 protein. *N* = 3 male mice per group

**Fig. 9:**
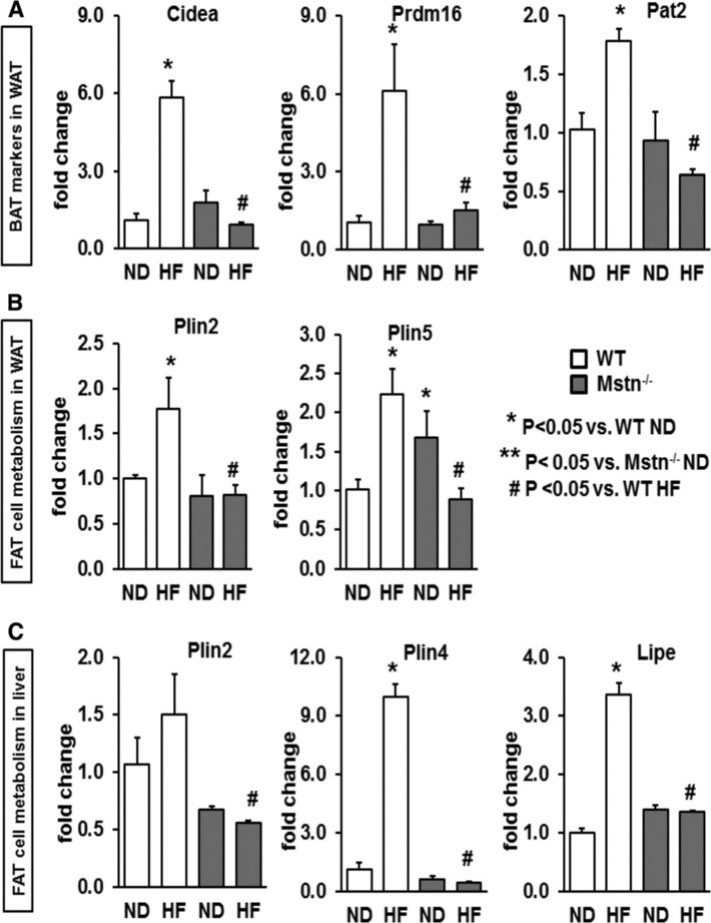
Gene expression of brown adipose tissue (BAT) markers and fat cell metabolism in **a**, **b** white adipose tissue (WAT) and **c** in the liver. ANOVA; (*) *P* < 0.05 vs. WT ND; (^#^) *P* < 0.05 vs. WT HF; (**) *P* < 0.05 vs. Mstn^−/−^ ND. *N* = 5 male mice per group

A qualitative evaluation of abdominal fat depots revealed a large increase in visceral fat contents in the Mstn^−/−^ mice in response to HF diet (Fig. 8a). This finding was unexpected given previous evidence suggesting that Mstn^−/−^ mice are protected against diet-induced obesity (e.g. [16, 17]). We sought to determine the gene expression patterns of the same key regulators of fatty acid uptake, oxidation, glucose metabolism and transcriptional regulators in white adipose tissue of WT and Mstn^−/−^ mice subjected to HF diet with a view of developing an understanding of mechanisms underpinning the excessive fat deposits in the Mstn^−/−^ mice fed on high-fat diet (Fig. 8b–d). A significant interaction between genotype and diet was evident for *Cpt1b*, *Slc25a20*, *Cpt2*, *Fatp1* and *Fabp3* originating in either a significant upregulation for the WT cohort or a blunted response for the Mstn^−/−^ mice (Fig. 8b). *Cd36* gene expression was induced in a similar manner for both genotypes. Similarly, fatty acid oxidation gene expression (i.e. *Acadl* and *Acadm* mRNA levels) was significantly upregulated in the WT mice and totally blunted in the Mstn^−/−^ mice (Fig. 8c). Taken together, these data suggest that fatty acid metabolism within the adipose tissue of the Mstn^−/−^ mice is transcriptionally compromised at least in two different levels; fatty acid uptake as well as fatty acid oxidation. On the contrary, WT mice gene expression changes suggest an increased capacity for both cellular uptake and use of fatty acids. Again, as for the liver, *Ppara* was more robustly activated in WT compared to Mstn^−/−^ in response to high fat (Fig. 8d). Examination of the expression of *Ucp1* in the fat revealed a striking finding. WT fat from mice raised on normal or high-fat diets expressed very little *Ucp1*. In contrast, Mstn^−/−^ mice expressed elevated levels of *Ucp1* in the normal state which was dramatically decreased following the introduction of a high-fat diet. Similar profiles were obtained for Ucp1 protein levels (Fig. 8c, e). These results suggest that the normal white fat of the Mstn^−/−^ mice has a high activity of *Ucp1* and thereby resembles brown fat. The browning of fat has been shown to be mediated by signalling initiated by *Fndc5*/*irisin* [32]. Recent studies have found that white fat expresses this protein and regulates its metabolic properties in an autocrine fashion [33]. We found that the expression of *Fndc5/irisin* decreased by 4-fold in WT mice following the introduction of a high-fat diet. In contrast, its levels dropped by 13-fold in Mstn^−/−^ mice following the same intervention (Fig. 8d). These results show that the fatty acid uptake and fatty acid oxidation programmes are robustly induced by high fat in adipose tissues of WT mice by high fat but this response is minimal in Mstn^−/−^ mice. Furthermore, we show that levels of *Ucp1* which would act to metabolise fat are dramatically decreased in the adipose tissue of Mstn^−/−^ mice in response to high fat possibly due to a decrease in the expression of *FNdc5*/*irisin.* Since the irisin-Ucp1 pathway that regulates the development of brown adipose tissue (BAT) was perturbed in Mstn^−/−^ HF mice, we measured transcript levels of key genes that promote BAT (i.e. *Cidea*, PR domain zinc finger protein 16; *Prdm16*; proton assistant amino acid transporter-2*, Pat2;* Fig. 9a). There was a significant increase of all three genes in WT HF mice, while Mstn^−/−^ HF mice showed a blunted response. Similarly, we measured the mRNA levels of *adipophilin* (*Plin2*) and *perilipin* (*Plin5*), two genes that regulate fat cell metabolism and lipid storage in white adipose tissue. Plin2 is known to have an adipogenic role, while Plin5 can be both adipogenic or lipolytic [34]. There was a significant increase in mRNA for both genes in WT but not in Mstn^−/−^ in response to HF (Fig. 9b). *Plin2* mRNA levels in the liver were not affected by diet, but there was a significant main effect of the genotype with Mstn^−/−^ HF mice having lower levels vs. WT cohorts. At last, we found a significant increase in *Plin4* and *Lipe* in the liver of WT HF mice without any change for the Mstn^−/−^ HF mice (Fig. 9c).

### Metabonomic analysis of skeletal muscle

**Fig. 10:**
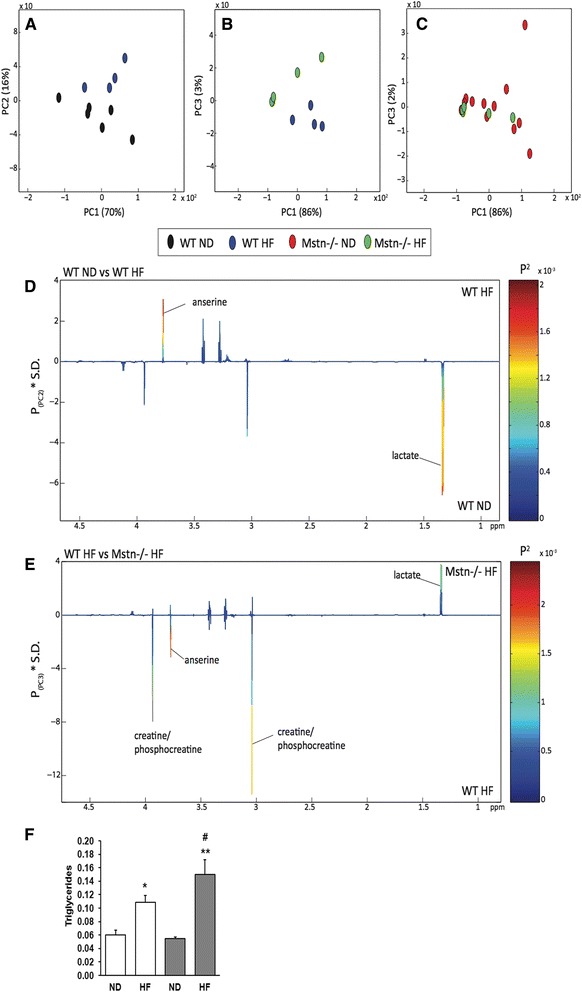
Metabonomic analysis of skeletal muscle. Pair-wise comparisons of the metabolic profiles obtained from gastrocnemius muscle from wild type (WT) and myostatin null (Mstn^−/−^) mice under normal and high-fat diet (ND and HF, respectively). Principal components analysis (PCA) scores plots comparing **a** WT ND vs. WT HF (PC1 vs. PC2); **b** WT HF vs. Mstn^−/−^ HF (PC1 vs. PC3); **c** Mstn^−/−^ ND vs. Mstn^−/−^ HF (PC1 vs. PC3); (% variance explained in the parenthesis). Colour loadings plots shown for **d** PC2 of the model comparing WT ND vs. WT HF and **e** PC3 of the model comparing WT HF vs. Mstn^−/−^ HF. Product of PC loadings with standard deviation of the entire data set coloured by the square of the PC loading. **f** Triglyceride levels in cellular extracts of the gastrocnemius muscle given as fractions. ANOVA; (*) *P* < 0.05 vs. WT ND; (^#^) *P* < 0.05 vs. WT HF; (**) *P* < 0.05 vs. Mstn^−/−^ ND. *N* = 4 male mice per group

Metabolic profiles were obtained from the hydrophilic extracts of the gastrocnemius muscle by ^1^H NMR spectroscopy. Score plot from the pair-wise PCA model comparing WT and Mstn^−/−^ control muscles revealed differences between the two genotypic groups driven by higher creatine/phosphocreatine in the muscles of WT mice and lower lactate compared to Mstn^−/−^ muscle (Fig. 10a–e). Clear differences were observed in the metabolic profiles of the muscles when feeding WT mice a HF diet (Fig. 10a). From the corresponding loadings plot, the HF diet can be seen to increase muscular anserine and decrease lactate in the WT mice (Fig. 10d). Anserine is a histidine-related compound with established antioxidants properties in skeletal muscle and brain of several species that is believed to inhibit lipid oxidation by means of free radical scavenging or metal chelation [35, 36]. In contrast, the HF diet did not induce any robust metabolic perturbations in the muscles of Mstn^−/−^ mice (Fig. 10b). Comparing the metabolic phenotypes of WT and Mstn^−/−^ muscle fed, a HF diet revealed the Mstn^−/−^ muscle to contain higher amounts of lactate while the WT muscle contained greater amounts of creatine/phosphocreatine and anserine (Fig. 10c). We complemented these studies by performing an extensive analysis in the lipid content in muscle. Triglyceride contents in cellular extracts from gastrocnemius showed a significant increase for both genotypes in response to HF diet, which however was more pronounced in KO HF mice (Fig. 10f). No major effects were observed for other lipid subclasses, except a significant decrease for phosphatidylethanolamines in HF mice for both genotypes (Additional file 3: Figure S2).

## Discussion

Previous evidence indicates that genetic loss of myostatin increases lean body mass, prevents adipose tissue accumulation and attenuates the obese and diabetic phenotypes in mice [17]. Since then, several groups have investigated whether the myostatin signalling inhibition can be an effective strategy against obesity and insulin resistance. It was proposed that inhibition of the myostatin pathway in mice results in resistance to develop high-fat diet-induced and genetic obesity, suggesting a potential role for myostatin inhibition in the treatment of obesity and diabetes (e.g. [37]). Intriguingly, our datasets provide comprehensive evidence against the protective role of myostatin deletion with regard to the development of obesity in the adult as well as perturbing the foetal muscle development programme following exposure to a high-fat diet. In particular, HF diet had devastating effects on both animal survival curves and transcriptional profiles of muscle, liver and fat tissue. These findings are in agreement with Guo et al. who reported increased fat mass and adipocyte size in Mstn^−/−^ mice held on a HF diet for 10 weeks [38]. However, on closer inspection of data rather than the headline statements there may be congruence between our finding and those of others who claim a protective effect as even Guo et al., state that Mstn^−/−^ ‘were not completely resistant to the effects of diet-induced obesity’ [38]. Certainly, our results are more pronounced than those previously reported and given that we have used the same lines as others, this suggests that environmental factors significantly influence the phenotype of mice with extremely physiological properties (hypermuscular, hyperglycolytic and fatigue prone). One influence could be the gut microbiota. This has been shown to vary across animal studies and even within wild-type mouse cohorts [39–41]. This is of particular relevance since the gut microbiome has been shown to regulate the activity of key molecules that control muscle lipid oxidation [42] and is able to protect against diet-induced obesity. We suggest that the microbiome of our mice differ from those in other research institutes. These variations have little effect on wild-type mice when challenged to a high-fat diet. However, Mstn^−/−^ mice which already display a genotype specific metabolic profile (including circulating lipids) respond to a high-fat diet in a detrimental manner and develop obesity. This line of thought could possibly be exploited to develop anti-obesity interventions by comparing the microbiome of a cohort of mice that are prone to fat deposition to others which are protective. Such a study, although very attractive is technically and logistically demanding and beyond the scope of this study.

Our data would argue against the beneficial role of myostatin deficiency in the control of obesity. However, antagonism of myostatin signalling towards the same end warrants further investigation, since epigenetic interventions, as opposed to germ line deletion, have been shown to increase muscle mass without major decreases in oxidation capacity [43]. However, there is a growing body of evidence that post-natal myostatin inhibition-mediated muscle growth has detrimental outcomes especially when the tissue is exposed to environmental stress. Two particular studies exemplify this fact. The study of Relizani showed that blockade of activin receptor IIB signalling induced muscle fatigability and metabolic myopathy [44]. Secondly, and of particular relevance to our study, is the finding of Wang et al. [45] who reported that post-natal inhibition of myostatin signalling in a type 1 diabetic model, rather than attenuating actually increased the severity of hyperglycemia. Indeed, the latter study demonstrated that myostatin inhibition led to elevated serum levels of corticosterone. Significantly, this class of glucocorticoid is known to promote obesity. We suggest that more studies in animal models are required before rolling out anti-myostatin treatments for either therapeutic or preventative regimes in humans.

We show that HF impacted on foetal muscle development more severely on the Mstn^−/−^ compared to wild-type mice, a conclusion reached by either comparing the number of muscles affected, decreases in fibre number as well as size and the higher number of immature fibres (gauged through central nucleation). HF has been shown to influence pre-natal muscle development by regulating the expression of key markers of myogenic commitment including MyoD, through local upregulation of NF-кB inflammatory signalling pathways [46]. We suggest that attenuated fat handling in maternal tissues in Mstn^−/−^ leads to increased lipid transfer across the placenta which is documented to cause widespread inflammation in obese conditions including the activation of NF-кB.

Skeletal muscle with high oxidative capacity and high prevalence of oxidative myofibres as demonstrated in various experimental models (e.g. transgenic mice overexpressing Ppard and ERRgamma) are associated with improved metabolic profiles and resistance to obesity [47, 48]. Thus, the predominant glycolytic, non-oxidative muscle phenotype found in Mstn^−/−^ (i.e. IIB fibres) would support the notion that they are susceptible in developing obesity. To gain an overview about the metabolic and contractile properties of the skeletal muscle under a HF diet, we assessed muscle fibre type, mitochondrial activity by means of SDH staining and contractile properties by measuring force. Our findings show that HF induced a shift towards more oxidative fibres (IIB to IIA) in EDL of the WT cohort. These changes were in accordance with more SDH positive fibres in EDL muscle. On the contrary, HF diet did not affect MHC and SDH in Mstn^−/−^ EDL muscle. Muscle oxidative properties are known to be impaired and mitochondrial DNA decreased in Mstn^−/−^ fast muscles [49] which could explain the blunted response of the Mstn^−/−^ EDL muscle.

### Metabolic gene expression changes in response to HF diet

Our molecular analysis suggests that WT mice exhibit a more robust transcriptional response in muscle to a high-fat diet compared to Mstn^−/−^. This finding suggests that Mstn^−/−^ mice can adapt their transcriptional machinery to uptake and utilize fatty acids in the skeletal muscle but do so sub-optimally. WT muscle responds to high fat not only by taking up lipids but also activating programmes promoting its disposal through the production of ATP evidenced by the induction of *Ucp1*. Despite these responses, the function of muscle in terms of tension production is compromised. Paradoxically, the blunted response of Mstn^−/−^ high fat protects it from fat-uptake induced muscle tension loss.

However, our study shows that other important fat handling tissues also malfunction in Mstn^−/−^ mice in response to high fat. Remarkably, whereas all the genes examined that control fatty acid uptake were upregulated by high fat in the livers of WT mice, none were affected by the intervention in Mstn^−/−^. It should be noted that *Ppara*, a master regulator was upregulated in both genotypes by high fat but again more robustly in WT liver. Most telling was our histological examination of the livers for fat storage in response to a change in diet. There was a huge increase in Oil Red O staining, an indicator of fat deposition, in the livers of WT mice but no change at all in the analogous tissues from Mstn^−/−^ mice. Hence, we suggest that the buffering capacity afforded by the liver in WT mice is negligible if not absent in Mstn^−/−^.

White adipose tissue serves as the primary lipid storage facility in the body. Furthermore, adipose tissue is able to adapt to increased available fat by increasing its rate of lipid oxidation thereby safeguarding against obesity [50]. However, high-fat diets have been demonstrated to not only leads to the hypertrophy of this tissue but also its dysfunction signified by the activation of stress pathways and the activation of macrophages leading to tissue remodelling [51]. Our results highlight two mechanisms which could act in concert to bring about the extreme levels of visceral adipose tissue found in the Mstn^−/−^ mice that were fed a high-fat diet. We report that markers of fatty acid oxidation (*Acad1* and *Acadm*) were upregulated in adipose tissue by the high-fat diet in WT mice but not changed in Mstn^−/−^ mice. Secondly, we show that the abnormally high levels of *Ucp1* and protein which would act to decrease fat content, found in normal diet Mstn^−/−^ tissue was dramatically reduced by the high-fat regime. Our finding that adipose tissue from Mstn^−/−^ mice displays elevated levels of *Ucp1* could explain the lack of fat in a number of species lacking the activity of this gene including mice, dogs, and humans [52–54]. Furthermore, they shed light onto the mechanisms by which Mstn^−/−^ displays elevated *Ucp1* expression through finding that the tissue expresses about 4-fold higher levels of *Fndc5/Irisin,* a mediator of fat browning. High-fat diet reduces the levels of this gene in Mstn^−/−^ to the levels found in WT tissue. Interestingly, we found that levels of *Fndc5* were unaffected in either background by diet in fast and slow muscle as well as liver (data not shown) implying that other myokines act to mediate the effect on adipose tissue reported here.

We suggest that the excessive visceral adipose that develops in Mstn^−/−^ induced by a high-fat diet is due to a blunting of the fatty acid oxidation programme and a decrease in mechanisms that dissipate oxidative energy as heat.

### Muscular metabolic response to HF diet

WT muscle undergoes metabolic adaptation in response to the HF diet, which is consistent with the oxidative phenotype of the muscle fibres that the WT animals possess. Reductions in tissue lactate in these mice reflect the preferential consumption of fatty acids as a primary substrate to support their energy requirements. Lactate is a product of anaerobic glycolysis, a process that is attenuated in the muscles of WT mice following a HF diet. In contrast, the Mstn^−/−^ mice appear unable to adapt to this dietary modulation. Indeed, comparing muscles from Mstn^−/−^ and WT mice fed a HF diet found the Mstn^−/−^ muscle to contain higher amounts of lactate and lower amounts of creatine/phosphocreatine. This biochemical variation results from the glycolytic phenotype of the Mstn^−/−^ muscle and the lack of oxidative capacity [55, 56]. Following a HF diet, anserine was observed to increase in the muscle of WT mice. Anserine is commonly found in the skeletal muscle of many vertebrates and has been shown to act as H^+^ buffer in glycolytic tissues [57], be an efficient metal-chelating agent [58] and activate myosin ATPase [59]. Anserine, and other histidine-related dipeptides, also possess antioxidant properties and protect against oxidative stress [36, 59]. Elevated anserine in the WT muscle may form part of a strategy to scavenge lipid oxidation by-products as reactive carbonyl species, formed during fatty acid oxidation, can react with DNA bases and lead to the formation of advanced lipoxidation products that can cause cellular damage and lead to oxidative stress-related diseases [60]. In agreement, there is evidence suggesting that histidine-containing dipeptides can quench reactive species originating from lipid oxidation in skeletal muscle [61].

From the metabolic profiles, the Mstn^−/−^ mice appear incapable of handling the metabolic consequences of a HF diet. Skeletal muscle of Mstn^−/−^ mice does respond transcriptionally to a high-fat diet but the expression of genes associated especially with fatty acid oxidation are severely attenuated which would offer a plausible explanation for the build-up of triglycerides that we detected through our lipidomic analysis.

## Conclusions

In the present study, we challenged the Mstn^−/−^ mouse by subjecting it to a high-fat diet regime for several weeks in an attempt to shed more light into the mechanistic insights of obesity development in this hypermuscular mouse model. Intriguingly, our data comprehensively demonstrates that myostatin deletion is not beneficial against the development of obesity and fat tissue accumulation. We show that skeletal muscle and liver of Mstn^−/−^ mice are unable to adapt in a normal manner to utilise excessive dietary fat. We suggest that this leads to accumulation in the adipose stores. However, at this site, the programme of fat oxidation is blunted leading to compartmental hypertrophy. Furthermore, we show that white adipose tissue of Mstn^−/−^ mice have brown fat characteristics exemplified by the elevated levels of *Fndc5*/*Irisin* and *Ucp1*. However, the levels of both these factors which would act to reduce adipose levels are greatly reduced by a high-fat diet. These results offer a novel explanation for the lean phenotype displayed by a range of animals lacking myostatin.

Finally, this work has evolutionary implications and offers an additional reason, to previous reports demonstrating hyperfatigability, as to why nature does not select a hypertrophic condition.

## Additional files

Additional file 1: Table S1. Blood parameters. Values from minimum of six male mice. (DOCX 13 kb) Additional file 2: Figure S1. Effect of high fat diet on Soleus muscle gene expression. Soleus gene expression levels of (A) Myostatin, (B) key factors regulating fatty acid uptake (Cpt1b, and Cpt2), (C) fatty acid oxidation (i.e. Acadl and Acadm) and (D) glucose metabolism (i.e. Glut1 and Glut 4). ANOVA; (*) P<0.05 vs. WT ND; (#) P<0.05 vs. WT HF; (**) P<0.05 vs. Mstn-/- ND. N=6 male mice per group. Additional file 3: Figure S2. Effect of high fat diet on Gastrocnemius lipid content. All values are fraction of total lipid content. ANOVA; (*) P<0.05 vs. WT ND; WT HF; (**) P<0.05 vs. Mstn-/- ND. N=6 male mice per group.

## Abbreviations

CSA: cross-sectional area
EDL: extensor digitorum longus
FID: free induction decay
HF: high fat
MHC: myosin heavy chain
Mstn: myostatin
NMR: nuclear magnetic resonance
PCA: principal component analysis
*P*_o_: maximum tetanic tension
*P*_t_: maximum twitch tension
RD: recycle delay
SDH: succinate dehydrogenase
SDS: special diet services
TSP: 3-(trimethylsilyl)-[2,2,3,3,-^2^H_4_]-propionic acid
Ucp1: uncoupling protein 1
WAT: white adipose tissue
WT: wild type
