# Supplementary material for: Investigating mechanisms underpinning the detrimental impact of a high-fat diet in the developing and adult hypermuscular myostatin null mouse
Source: Skelet Muscle. 2015 Dec 7;5:38. doi: 10.1186/s13395-015-0063-5 (PMC4671215; doi:10.1186/s13395-015-0063-5)
Supplement: Additional file 1: Table S1. — Blood parameters. Values from minimum of six male mice. (DOCX 13 kb) [file 13395_2015_63_MOESM1_ESM.docx]

Table S1. Blood parameters

|  | Wild type | | Mstn^-/-^ | |
| --- | --- | --- | --- | --- |
|  | **Normal diet** | **High fat diet** | **Normal diet** | **High fat diet** |
| Urea (mmol/l) | 8.27±0.83 | 10.25±0.87 | 12.03±0.12 | 10.03±0.38 |
| Creatinine (μmol/l) | 8.13±1.21 | 9.45±1.05 | 12.53±1.23 | 12.40±0.10 |
| Total protein (g/l) | 53.80±2.92 | 57.25±1.60 | 58.30±0.10 | 59.67±1.53 |
| Albumin (g/l) | 28.20±1.51 | 28.85±1.21 | 31.03±2.52 | 31.50±1.13 |
| Alkaline phosphatase (U/l) | 103.67±9.71 | 64.25±10.01 | 105.33±3.79 | 43.00±3.61 |
| Alanine aminotransferase (U/l) | 58.33±4.51 | 37.75±14.29 | 74.00±4.36 | 25.33±2.52 |
| Aspartate aminotransferase (U/l) | 154.00±32.60 | 163.00±50.73 | 99.00±1.00 | 159.33±5.13 |
| Total bilirubin (μmol/l) | 3.40±1.28 | 2.68±0.21 | 1.50±0.10 | 4.30±0.10 |
| Total cholesterol (mmol/l) | 2.75±0.53 | 5.11±0.17 | 2.94±0.14 | 5.14±0.05 |
| HDL cholesterol (mmol/l) | 1.49±0.26 | 3.41±0.07 | 1.90±0.05 | 3.63±0.36 |
| LDL cholesterol (mmol/l) | 0.79±0.19 | 1.58±0.12 | 0.84±0.03 | 1.80±0.10 |
| Triglycerides (mmol/l) | 1.20±0.37 | 2.04±0.54 | 2.14±0.04 | 2.99±0.05 |
| Glycerol (μmol/l) | 293.00±37.72 | 435.00±21.32 | 182.00±11.53 | 416.67±6.81 |
| Free fatty acids (mmol/l) | 0.65±0.03 | 0.63±0.13 | 0.25±0.03 | 0.76±0.06 |
| Fructosamine (μmol/l) | 225.67±20.01 | 229.75±10.05 | 268.67±5.13 | 238.67±10.26 |
| Glucose (mmol/l) | 13.42±0.99 | 14.16±2.46 | 13.27±1.10 | 15.30±0.61 |
| LDH (U/l) | 600.67±85.63 | 646.50±106.32 | 383.67±14.57 | 1580.67±105.66 |
| Amylase (U/l) | 771.67±94.88 | 872.67±5.03 | 1086.00±18.03 | 1343.00±151.88 |
| Creatine kinase (U/l) | 885.67±134.03 | 790.50±326.74 | 336.00±10.00 | 1324.33±56.58 |
| Ketone bodies (mmol/l) | 0.07±0.00 | 0.07±0.01 | 0.08±0.01 | 0.06±0.01 |
