# Supplementary figures and images for: Investigating mechanisms underpinning the detrimental impact of a high-fat diet in the developing and adult hypermuscular myostatin null mouse
Source: Skelet Muscle. 2015 Dec 7;5:38. doi: 10.1186/s13395-015-0063-5 (PMC4671215; doi:10.1186/s13395-015-0063-5)

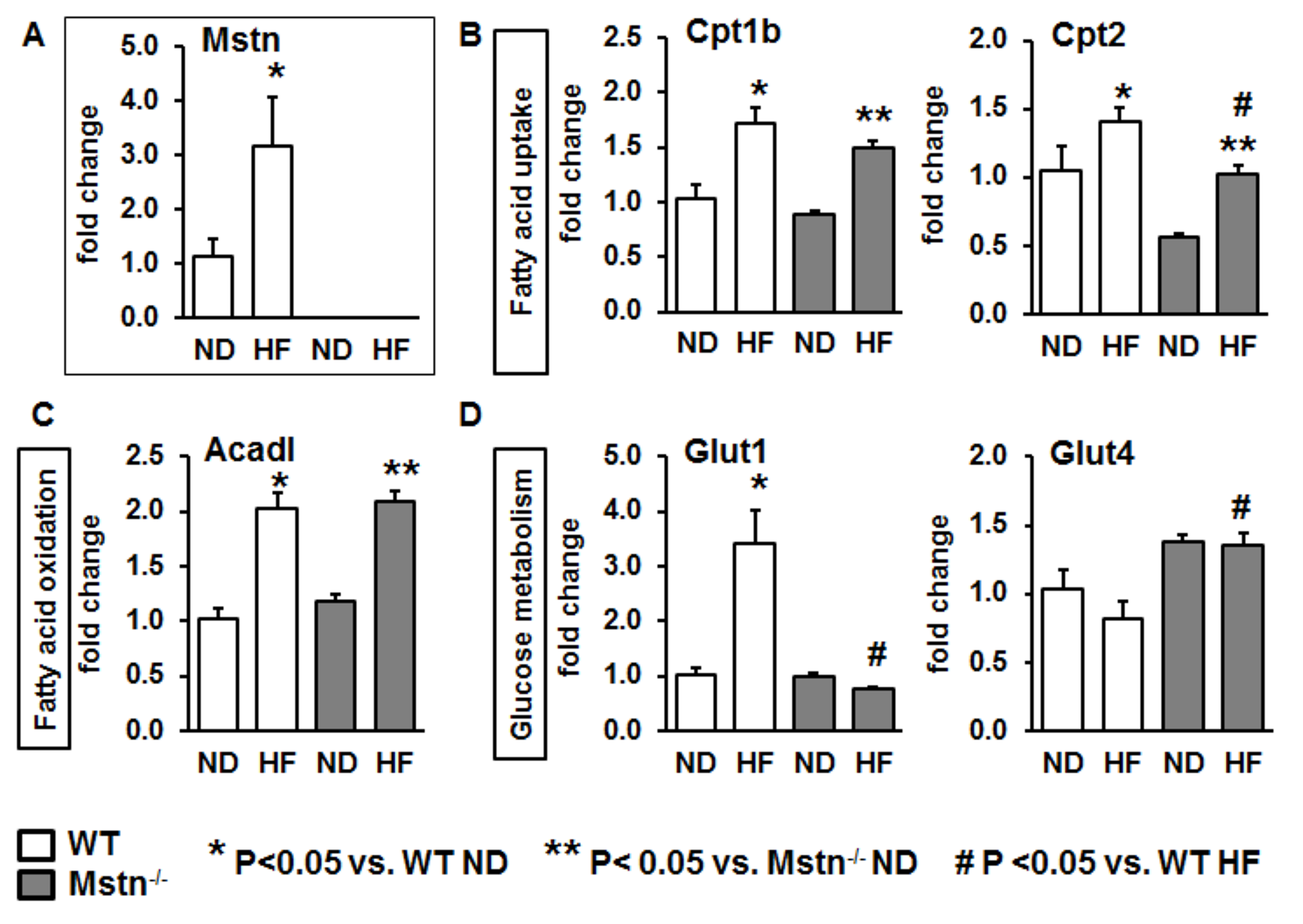

Supplement: Additional file 2: Figure S1. — Effect of high fat diet on Soleus muscle gene expression. Soleus gene expression levels of (A) Myostatin, (B) key factors regulating fatty acid uptake (Cpt1b, and Cpt2), (C) fatty acid oxidation (i.e. Acadl and Acadm) and (D) glucose metabolism (i.e. Glut1 and Glut 4). ANOVA; (*) P<0.05 vs. WT ND; (#) P<0.05 vs. WT HF; (**) P<0.05 vs. Mstn-/- ND. N=6 male mice per group. [file 13395_2015_63_MOESM2_ESM.tiff]

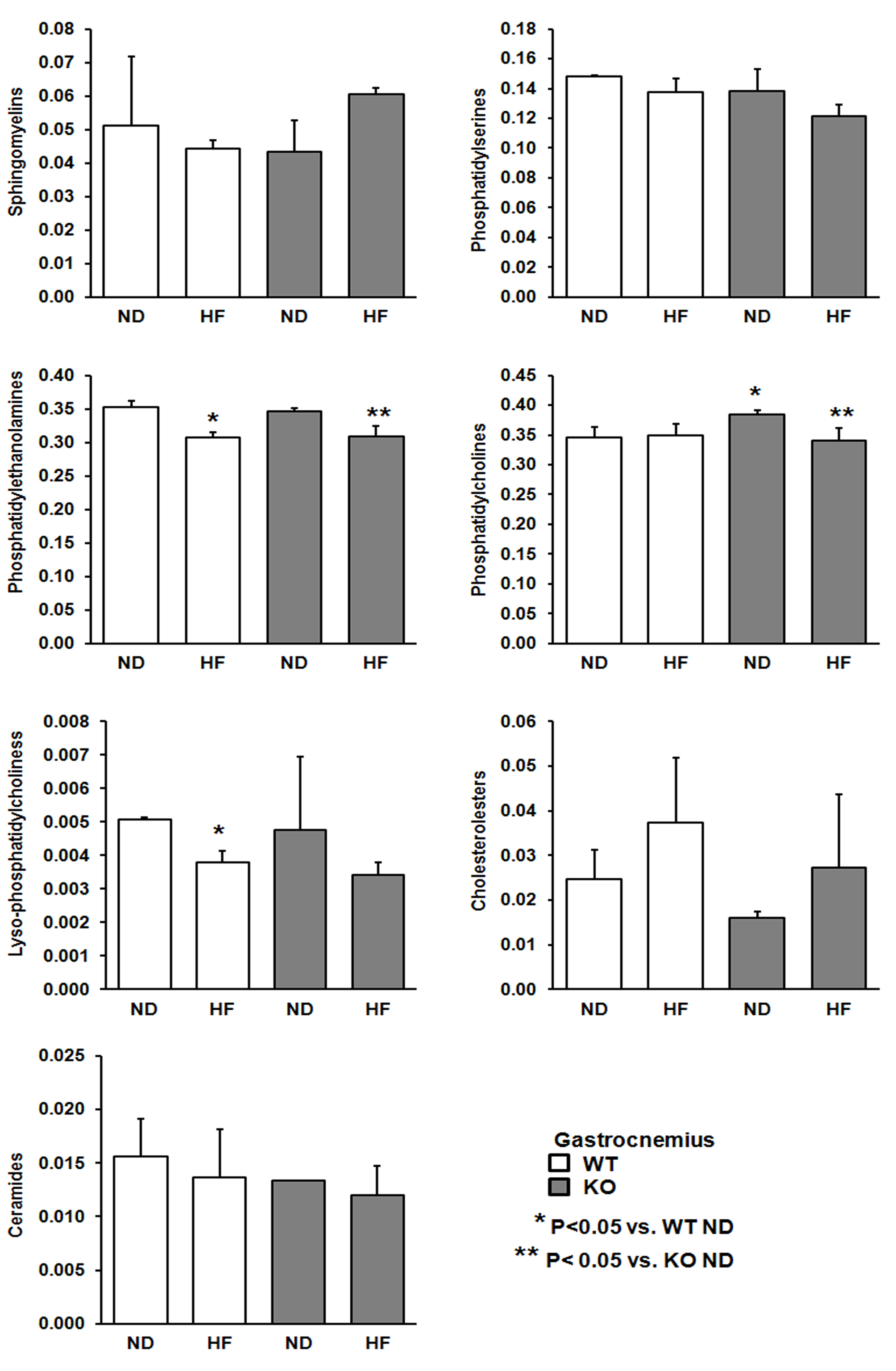

Supplement: Additional file 3: Figure S2. — Effect of high fat diet on Gastrocnemius lipid content. All values are fraction of total lipid content. ANOVA; (*) P<0.05 vs. WT ND; WT HF; (**) P<0.05 vs. Mstn-/- ND. N=6 male mice per group. [file 13395_2015_63_MOESM3_ESM.tiff]
